# Influence of Single-Nucleotide Polymorphisms in PPAR-δ, PPAR-γ, and PRKAA2 on the Changes in Anthropometric Indices and Blood Measurements through Exercise-Centered Lifestyle Intervention in Japanese Middle-Aged Men

**DOI:** 10.3390/ijms19030703

**Published:** 2018-03-01

**Authors:** Yuichiro Nishida, Minako Iyadomi, Hirotaka Tominaga, Hiroaki Taniguchi, Yasuki Higaki, Hiroaki Tanaka, Mikako Horita, Chisato Shimanoe, Megumi Hara, Keitaro Tanaka

**Affiliations:** 1 Department of Preventive Medicine, Faculty of Medicine, Saga University, Saga 849-8501, Japan; horitam@cc.saga-u.ac.jp (M.H.); chisatos@cc.saga-u.ac.jp (C.S.); harameg@cc.saga-u.ac.jp (M.H.); tanakake@cc.saga-u.ac.jp (K.T.); 2 SUMCO Corporation, Saga 849-4271, Japan; miyadomi@sumcosi.com; 3 Section of Clinical Cooperation System, Center for Comprehensive Community Medicine, Faculty of Medicine, Saga University, Saga 849-8501, Japan; hirotaka@cc.saga-u.ac.jp; 4 Institute of Genetics and Animal Breeding of the Polish Academy of Sciences, 05-552 Jastrzebiec, Poland; h.taniguchi@ighz.pl; 5 Laboratory of Exercise Physiology, Faculty of Health and Sports Science, Fukuoka University, Fukuoka 814-0180, Japan; higaki@fukuoka-u.ac.jp (Y.H.); htanaka@fukuoka-u.ac.jp (H.T.)

**Keywords:** exercise, PPAR, SNP, obesity, lipid, glucose, HbA_1c_, liver enzyme

## Abstract

The purpose of the current study was to examine the influence of single-nucleotide polymorphisms (SNPs) in the peroxisome proliferator-activated receptor-δ (PPAR-δ), PPAR-γ, and α2 isoforms of the catalytic subunit of AMP-activated protein kinase (PRKAA2) on the extent of changes in anthropometric indices and blood measurements through exercise-centered lifestyle intervention in middle-aged men. A total of 109 Japanese middle-aged male subjects (47.0 ± 0.4 years) participated in the baseline health checkup, 6-month exercise-centered lifestyle intervention, and second checkup conducted several months after the subject completed the intervention. The body mass index (BMI), waist circumference, and clinical measurements, including hemoglobin A_lc_ (HbA_1c_), triglyceride (TG), alanine aminotransferase (ALT), and γ-glutamyl-transpeptidase (γ-GTP), were measured at the baseline and second checkup. The three SNPs of PPAR-δ A/G (rs2267668), PPAR-γ C/G (rs1801282), and PRKAA2 A/G (rs1418442) were determined. Blunted responses in the reduction in the BMI and waist circumference were observed in A/A carriers of PPAR-δ SNP compared with G allele carriers (all *p* < 0.05). The A/A carriers also displayed less-marked improvements in HbA_1c_, TG, ALT, and γ-GTP (all *p* < 0.05). The current results suggest that A/A carriers of PPAR-δ SNP (rs2267668) may enjoy fewer beneficial effects of exercise-centered lifestyle intervention on anthropometric indices and blood measurements.

## 1. Introduction

Peroxisome proliferator-activated receptors (PPARs) are nuclear receptors that play an important role in obesity and metabolism [1,2]. There are three isoforms of PPARs—PPAR-α, PPAR-δ/β, and PPAR-γ—all of which are activated by dietary or endogenous fatty acids and their metabolic derivatives and regulate lipid and glucose metabolism by modulating the expression of their target genes [1,2]. While PPAR-α and PPAR-γ are preferentially expressed in liver and adipose tissue, respectively [1], PPAR-δ is ubiquitously expressed throughout the body, and its expression is especially high in skeletal muscle compared with the other two isoforms [3,4]. These three PPAR isoforms together control various aspects of fatty acid metabolism, energy balance, insulin sensitivity, and glucose homeostasis through their coordinated activities in adipose tissue, liver, and skeletal muscle [1]. Another key molecule in lipid and glucose metabolism as well as exercise adaptation is AMP-activated protein kinase (AMPK). AMPK is a heterotrimeric protein composed of a catalytic subunit (α) and regulatory subunits (β and γ), and the catalytic subunit α has two isoforms (α1 and α2) [5]. While no defects in glucose homoeostasis were observed in AMPK α1 knockout mice, AMPK α2 knockout mice were insulin-resistant [6].

There have been several reports regarding the influence of single-nucleotide polymorphisms (SNPs) in PPARs on changes in anthropometric and metabolic parameters following lifestyle interventions in Caucasians [7,8]. Thamer et al. [8] showed that G allele carriers of the A/G SNP in PPAR-δ gene (rs2267668) displayed reduced responses to a nine-month exercise and dietary lifestyle intervention, as they lost less adipose tissue mass, less hepatic lipid content, and had less improvement in insulin sensitivity following the lifestyle intervention than A/A genotype carriers. Similarly, it has also been reported that the G allele carriers of the PPAR-δ A/G enjoy fewer benefits than A/A genotype carriers regarding insulin sensitivity after nine-month exercise and dietary intervention [7]. Several studies have also shown associations of the Pro12Ala variant of PPAR-γ (rs1801282) with obesity, type 2 diabetes/insulin resistance, and metabolic syndrome [9,10,11,12,13], and the PRKAA2 SNP (rs1418442) has also been reported to be associated with serum cholesterol levels in Caucasians [14] and with type 2 diabetes in Japanese populations [2,15].

Gene-exercise interaction is influenced by many factors of exercise regimens (e.g., exercise type, frequency, duration) and subject characteristics (e.g., ethnicity, age, sex, energy intake, and baseline physical activity) [16]. Thus, the currently available evidence regarding the influence of gene polymorphisms on the responsiveness to exercise intervention is far from sufficient to develop tailored exercise interventions based on an individual’s genetic information. In the current study, we selected the SNP PPAR-δ rs2267668 whose influence has previously been investigated in the aforementioned lifestyle intervention studies in Caucasians [7,8]. In addition, we also selected two other SNPs that are most well-known as metabolism-related SNPs (PPAR-γ rs1801282, PRKAA2 rs1418442) in Caucasians and Japanese [2,9,10,11,12,13,15]. Thus, the current study investigated the influence of these SNPs on the extent of changes in anthropometric indices and clinical blood measurements in response to six-month exercise-centered lifestyle intervention in Japanese middle-aged subjects.

## 2. Results

The genotype distributions of the three SNPs, PPAR-δ A/G (rs2267668), PPAR-γ C/G (rs1801282), and PRKAA2 A/G (rs1418442) in the current participants did not deviate from the Hardy-Weinberg equilibrium (*p* > 0.05). These genotype distributions were confirmed to be similar to those reported in a public database dbSNP (HapMap-JPT). The baseline levels of variables, such as the age, body mass index (BMI), waist circumference, systolic blood pressure (SBP), diastolic blood pressure (DBP), and clinical blood measurements (plasma glucose, hemoglobin A_lc_ (HbA_1c_), triglyceride (TG), high-density lipoprotein cholesterol (HDL-C), low-density lipoprotein cholesterol (LDL-C), total cholesterol (Total-C)), were not significantly different among the genotypes of the three SNPs (Table 1).

In all 109 subjects, the anthropometric indices of the BMI and waist circumference, and blood measurements such as TG, aspartate aminotransferase (AST), alanine aminotransferase (ALT), HDL-C, and total-C were significantly improved after the exercise-centered lifestyle intervention (Table 2). The decreases in the body weight, BMI, and waist circumference were significantly less marked in the A/A genotype carriers of PPAR-δ A/G (rs2267668) than in the G allele carriers (A/G + G/G) (Table 2). Similarly, the PPAR-δ A/A carriers displayed significantly less-marked improvements in the HbA_1c_, TG, ALT, and γ-glutamyl-transpeptidase (γ-GTP) (Table 2). However, no significant differences among genotype groups were observed in the other two tested SNPs (Table 2). The post-intervention values of variables measured at the second checkup are shown in Supplementary Table S1.

Univariate correlation analyses showed that, in the total subjects (*n* = 109), a greater reduction in the body weight was significantly correlated with greater improvements (decreases) in the HbA_1c_ (*r* = 0.24, *p* < 0.05), TG (*r* = 0.42, *p* < 0.01), ALT (*r* = 0.44, *p* < 0.01), and γ-GTP (*r* = 0.40, *p* < 0.01). The correlation analyses performed separately by the genotype groups of PPAR-δ SNP (A/A (*n* = 66) and A/G + G/G (*n* = 43)) showed a similar pattern of correlation (data not shown). Multiple linear regression analyses showed that the associations of the PPAR-δ SNP with the changes in HbA_1c_ (*p* = 0.26) and ALT (*p* = 0.31) were attenuated, being statistically insignificant, whereas the associations of the PPAR-δ SNP with the changes in TG (natural log-transformed) (*p* = 0.02) and γ-GTP (*p* = 0.04) remained significant even after adjustment for the change in body weight (Table 3).

## 3. Discussion

In the current study, we investigated the influence of PPAR-δ A/G (rs2267668), PPAR-γ C/G (rs1801282), and PRKAA2 A/G (rs1418442) SNPs on the anthropometric and blood measurements in response to exercise-centered lifestyle intervention in Japanese middle-aged men. The major finding of the current study was that the A/A homozygotes of PPAR-δ rs2267668 had less marked weight loss after the intervention than the G allele carriers. Similarly, the A/A carriers also displayed less-marked improvements in clinical measurements, such as the values of HbA_1c_, TG, ALT, and γ-GTP. Multiple linear regression analyses showed that the associations of the PPAR-δ SNP with the changes in HbA_1c_ and ALT were attenuated, being statistically insignificant, whereas the associations with TG and γ-GTP remained significant even after adjustment for changes in the body weight. These findings therefore suggest that the reduced improvements in TG and γ-GTP might be due to the A/A genotype itself, rather than the blunted weight loss response. In contrast, the other two tested SNPs in PPAR-γ and PRKAA2 did not influence the changes in the measured parameters.

PPAR-δ plays key roles in lipid and glucose metabolism and skeletal muscle adaptation to exercise [5]. In rodent studies, transgenic activation of PPAR-δ in adipose tissue has been shown to promote fatty acid oxidation in skeletal muscle and adipose tissue, preventing obesity [17], while PPAR-δ-deficient mice receiving a high-fat diet have reduced energy uncoupling and are prone to obesity [17]. Previous human studies have investigated the influence of the PPAR-δ A/G (rs2267668) SNP on the body fat reduction response and glucose metabolism indices after lifestyle intervention in Caucasians [7,8]. In these studies, lifestyle intervention-induced improvements (reductions) in adiposity and hepatic fat storage were blunted in G allele carriers of this SNP [8]. In contrast, in the current study A/A homozygous carriers of the PPAR-δ A/G (rs2267668) SNP displayed less-marked effects in anthropometric indices and clinical blood measurements than G allele carriers after six-month lifestyle intervention.

The reason for this discrepancy in findings between the previous reports and the present study is unclear. It may be due in part to differences in the exercise regimens (intensity, frequency, duration) and subject characteristics, such as the sex, age, energy intake, and baseline physical activity [16]. In addition, differences in the genetic backgrounds of European and Japanese subjects may be another possible reason, as the influence of gene polymorphisms on the degree of benefit from lifestyle intervention may not necessarily be the same (or may even be contrary) between different ethnic groups [16,18,19,20], although the reasons for the race-related differences in the influence of gene polymorphisms remain to be explored.

The physiological function of PPAR-δ A/G SNP (rs2267668) is unclear at present. However, one report evaluated the impact of this SNP (rs2267668) on the gene expression of PPAR-δ in human skeletal muscle [21]. This previous study showed that the A/A genotype carriers of PPAR-δ A/G SNP (rs2267668) had lower PPAR-δ mRNA expression in skeletal muscle than G allele carriers [21]. It is, therefore, biologically plausible that reduced PPAR-δ transcriptional activity in the muscle of A/A genotype carriers might be a reason for their blunt responses to exercise-centered health guidance intervention.

Less-marked decreases in the body weight and waist circumference observed in the A/A genotype of PPAR-δ A/G (rs2267668) SNP than in G allele carriers may have been due to insufficient increases in energy expenditure by physical activity and/or insufficient decreases in energy intake through the diet. We were unable to clarify which factors might have contributed most strongly to the blunted responses observed in the A/A genotype carriers. A rodent study found that treatment with a PPAR-δ agonist (GW50516) significantly retards weight gain but does not affect food consumption [1,22]; given those findings, we speculate that our subjects with the A/A genotype of PPAR-δ A/G (rs2267668) SNP may have expended less energy (rather than having a higher energy intake) than G allele carriers during the exercise-centered lifestyle intervention.

The major limitation of the current study was that we did not have precise data on the duration of exercise training performed by the subjects, although we instructed our subjects to regularly perform cycle ergometer exercise or brisk walking at the lactate threshold (LT) intensity, with a goal of >140 min/week. Second, we did not assess the effects of the exercise-centered lifestyle intervention immediately after completing the six-month intervention, instead evaluating the outcomes at the second checkup, which was conducted several months after the completion of the intervention. We were unable to clarify whether the reduced effects of the exercise-centered lifestyle intervention observed in the A/A genotype carriers of the PPAR-δ A/G SNP might have been due to less-marked changes in the measured parameters or an earlier return towards baseline values. We did not instruct the current subjects to maintain (not to change) their dietary habit during the exercise-centered lifestyle intervention period. We therefore cannot exclude the possibility that the subjects might have changed their dietary habits during the study period. We also did not collect any data on the eating habits of the study participants nor on their post-intervention habits. Although using bioelectric impedance or computed tomography to assess the body composition or abdominal/ectopic fat accumulation is ideal, we merely assessed the anthropometric indices (weight, BMI, waist circumference) included as inspection items for the annual specific health checkup. In addition, the current results were obtained in a Japanese population and cannot be generalized to other ethnic groups, such as Caucasians.

In conclusion, the current results suggest that the A/A genotype carriers of the PPAR-δ A/G SNP (rs2267668) may have experienced blunted effects in anthropometric measurements (weight, BMI, waist circumference), HbA_1c_, TG, and serum liver enzymes (ALT, γ-GTP) through exercise-centered lifestyle intervention compared with G allele carriers among our population of middle-aged Japanese men. In addition, multiple linear regression analyses suggested that the less-marked improvements in TG and γ-GTP observed in the PPAR-δ SNP A/A carriers were not due to their attenuated weight reduction. Studying the genetic background of multifactorial processes, such as weight loss, is challenging. In the present study, we were unable to clarify the extent to which the observed results depended on the genetic predisposition or difference in the subjects’ compliance to the exercise-centered lifestyle intervention. Therefore, a further study on the influence of PPAR-δ A/G SNP (rs2267668) is needed, in which exercise intervention is standardized and its compliance is carefully monitored. The current results will aid in the development of tailored health guidance programs based on the individuals’ genotypes for metabolism-related SNPs.

## 4. Materials and Methods

### 4.1. Subjects

The subjects were 109 Japanese middle-aged men (47.0 ± 0.4 years of age) employed at a silicon wafer manufacturer (Saga, Japan) who participated in the baseline (first) checkup (conducted as an annual specific health checkup), 6-month exercise-centered lifestyle intervention (conducted as a specific health guidance), and second checkup conducted 1 year after the baseline health checkup. The post-intervention assessments at the second checkup were conducted several months after each subject completed the six-month intervention. The specific health checkup (to identify individuals with metabolic syndrome) and the specific health guidance (to improve their metabolic syndrome) are currently conducted as part of a national effort against metabolic syndrome, in which medical insurers are obligated to provide insured middle-aged employees (40–74 years of age) with specific health checkups. Participants are recommended to participate in the specific health guidance if they meet certain conditions of metabolic syndrome (Available online: http://www.mhlw.go.jp/english/wp/wp-hw3/dl/2-007.pdf).

The inclusion criteria of the specific health guidance for men were as follows: abdominal obesity (waist circumference ≥ 85 cm) plus at least 1 of the following 3 components: (1) dyslipidemia (TG ≥ 150 mg/dL and/or HDL < 40 mg/dL); (2) high blood pressure (SBP/DBP ≥ 130/85 mm Hg); and (3) high blood glucose (fasting plasma glucose ≥ 100 mg/dL or HbA_1c_ expressed as National Glycohemoglobin Standardization Program (NGSP) value of 5.6%). In addition, even if the waist circumference was <85 cm, men who had a BMI ≥ 25 kg/m^2^ plus at least 1 of the 3 abovementioned components met the requirements to participate in the specific health guidance. Subjects who were taking medications for type 2 diabetes, hypertension, or dyslipidemia were excluded.

### 4.2. Anthropometric and Blood Measurements at the Baseline (First) Checkup and Second Checkup (Conducted as Annual Specific Health Checkup)

The BMI was determined by dividing the body weight in kilograms by the square of the height in meters. The waist circumference was measured at the level of the umbilicus. Blood pressure (SBP and DBP) was measured in the sitting position after 5 min of rest using an automatic sphygmomanometer. For the blood biochemical test, blood samples were obtained from an antecubital vein after an overnight fast. Plasma glucose was measured via the standard method. HbA_1c_ was measured by a latex aggregation immunoassay (Japan Diabetes Society (JDS) value). The HbA_1c_ was estimated as a NGSP equivalent value calculated by the formula as follows: HbA_1c_ (NGSP (%)) = 1.02 × HbA_1c_ (JDS (%) + 0.25% [23]. Total-C and TG levels were measured enzymatically. HDL-C and LDL-C were measured via direct methods. Serum liver enzymes (AST, ALT, γ-GTP) were also measured by standard methods. The ectopic fat accumulation in the liver is an important factor for inducing insulin resistance [24], and serum liver enzymes, especially ALT, can be practical indices reflective of the liver fat accumulation [25]. All measurements and blood biochemical analyses were performed using similar methods at the baseline and second checkup.

### 4.3. Six-Month Exercise-Centered Lifestyle Intervention (Conducted as Specific Health Guidance)

First, an initial group-based health guidance (≤8 participants/group for 80 min) was conducted, in which the subjects received an explanation on the results of their baseline checkup from public health nurses and then set achievable goals related to lifestyle improvement with support or advice from public health nurses. The subjects were instructed to record information on whether their own-set goals were achieved on a recording sheet (made using the Excel software program). Throughout the six-month intervention, the subjects received personalized follow-up consultation through e-mail.

The main components of the 6-month exercise-centered intervention were two sessions of group-based (≤8 participants/group) exercise guidance (90 min/session). In the first exercise session, a submaximal graded exercise test using a cycle ergometer (Model EC-3600; Cateye Inc., Osaka, Japan) was performed to assess the lactate threshold (LT) as an index of aerobic capacity, and moderate exercise at the LT intensity was recommended to the subjects as an ideal exercise for their daily exercise training. For the graded exercise test, the work rate was initially set at a workload corresponding to 3 metabolic equivalents (METs) and then increased by 1 MET every 3 min (i.e., 3, 4, 5, 6, and 7 METs). Oxygen consumption was estimated based on the workload and subjects’ body weight using the American College of Sports Medicine leg ergometer equation, as follows: the estimated oxygen consumption (mL/kg/min) = workload (watts) × 6.12 × 1.8/body weight (kg) + 7 [26]. The METs were calculated as the estimated oxygen consumption divided by 3.5. The end-point of the exercise test was determined based on either achieving a blood lactate concentration of 4 mM or the American College of Sports Medicine criteria [27]. The heart rate was measured in real time using a sensor (installed in the cycle ergometer) attached to the earlobe. The heart rate and rating of perceived exertion (RPE) [28] were recorded every 3 min during the tests. Blood samples (5 μL) were also obtained from the earlobe every 3 min to measure the blood lactate concentration using a portable blood lactate test meter (Lactate Pro; ARKRAY, Inc., Kyoto, Japan).

The blood lactate concentration (mM) was plotted against the exercise workload (watts) for each subject. The estimated oxygen consumption (or METs) at the first breakpoint of lactate concentration was used as the data of LT. The LT is a reliable indicator of aerobic fitness that is in no way inferior to VO_2_ max [29,30], and this index can be simply and precisely measured by a graded cycle ergometer test using a portable lactate analyzer [20]. Immediately after the cycle ergometer exercise test, we provided an exercise prescription based on each subject’s result for the cycle ergometer exercise test, and the subjects were instructed to perform cycle ergometer training at the LT intensity and/or brisk walking at a heart rate corresponding to the LT, with a goal of ≥140 min/week. The factories of the subjects’ employer include training rooms (in which there were many cycle ergometers and treadmills) that were freely available to all subjects during their rest period or after working hours.

Two months after the first session of exercise guidance, the second session of exercise guidance (≤8 participants/group for 90 min) was conducted, mainly to revise the exercise intensity and to maintain or promote subjects’ motivation to continue the cycle ergometer exercise and/or brisk walking at the LT intensity. In this second session of exercise guidance, the subjects performed a single bout of 15-min cycle ergometer exercise at the LT that was previously determined at the first session of exercise guidance, and the heart rate was monitored during the exercise to assess if the aerobic capacity had improved. The heart rate during the exercise was used to give feedback to the subjects. The subjects received individual advice on the exercise training based on the newly revised workload. Within one month after completing the six-month intervention, the body weight, waist circumference, and blood pressure were assessed, but these data were not collected for the current study, as this time point was not an endpoint of our study.

### 4.4. Genotyping of Gene Variants

Genomic DNA was extracted from saliva (2 mL) collected from subjects using Oragene DNA kits (DNA Genotek, Ottawa, ON, Canada). DNA was purified from 200-μL aliquots of Oragene DNA/saliva samples using an ethanol precipitation protocol supplied with the kits. Purified DNA was redissolved in 200 μL of Tris ethylenediaminetetraacetic acid buffer (10 mM tris-HCl, 1 mM ethylenediaminetetraacetic acid, pH 8.0). The SNPs in PPAR-δ A/G rs2267668 (located in intron 2), PPAR-γ C/G rs1801282 (exon 1 (Pro12Ala), and PRKAA2 A/G rs1418442 (intron 4) were analyzed by a TaqMan^®^ SNP Genotyping Assay using a StepOne Plus real-time PCR system (Applied Biosystems, Foster City, CA, USA). Their assay IDs were C__5872729_10 for PPAR-δ A/G (rs2267668), C__1129864_10 for PPAR-γ C/G (rs1801282)), and C__2821517_20 for PRKAA2 A/G (rs1418442).

### 4.5. Statistical Analyses

Values were shown as the mean ± standard error (SE) or the median (interquartile range). The chi-squared test was used to confirm whether the genotype frequencies were in Hardy-Weinberg equilibrium. Between-group (between genotype groups) comparisons were performed using Welch’s *t*-test for the age, weight, BMI, waist circumference, SBP, DBP, glucose, HbA_1c_, HDL-C, LDL-C, Total-C, and LT. Since the distributions of TG, AST, ALT, and γ-GTP may have been skewed, between-group comparisons of these four variables were performed using a nonparametric Wilcoxon’s signed-rank test. Within-group comparisons between the baseline and second checkup were also performed using Wilcoxon’s signed-rank test. The significance of correlations between two variables was assessed using Pearson’s correlation coefficient. Multiple linear regression analyses with adjustment for change in body weight were performed to examine whether the observed significant differences in the changes in the clinical blood measurements between genotypes were independent of the change in the body weight. In these multiple linear regression analyses, PPAR-δ A/G SNP (rs2267668) was used as an independent variable, and the changes in four clinical measurements (HbA_1c_, natural log-transformed TG, ALT, γ-GTP) were used as dependent variables, with the change in body weight used for adjustment. The statistical analyses were performed using the R software program (version 3.4.1). *p* Values of <0.05 were considered statistically significant.

## Figures and Tables

**Table 1:** Baseline characteristics of the subjects according to genotypes of PPAR-δ, PPAR-γ, and PRKAA2 SNPs.

Variables	All Subjects (*n* = 109)			PPAR-δ rs2267668							PPAR-γ rs1801282							PRKAA2 rs1418442						
				A/A (*n* = 66)			A/G + G/G (*n* = 43)			*p* Value *	C/C (*n* = 99)			C/G + G/G (*n* = 10)			*p* Value *	A/A (*n* = 64)			A/G + G/G (*n* = 45)			*p* Value *
Age (years)	47.0	±	0.4	46.8	±	0.5	47.3	±	0.6	0.61	47.1	±	0.4	46.1	±	1.3	0.49	47.1	±	0.5	46.9	±	0.7	0.78
Weight (kg)	75.3	±	0.8	76.1	±	1.0	74.2	±	1.2	0.24	75.0	±	0.8	79.1	±	2.9	0.20	75.6	±	1.0	74.9	±	1.2	0.67
BMI (kg/m^2^)	25.7	±	0.2	25.8	±	0.3	25.5	±	0.4	0.64	25.5	±	0.2	27.2	±	0.6	0.02	25.8	±	0.3	25.6	±	0.3	0.67
Waist circumference (cm)	90.0	±	0.5	90.3	±	0.7	89.5	±	0.8	0.47	89.7	±	0.5	93.3	±	2.2	0.15	90.5	±	0.7	89.4	±	0.7	0.29
SBP (mmHg)	125	±	1	126	±	1	122	±	2	0.08	125	±	1	125	±	2	0.85	125	±	1	124	±	2	0.46
DBP (mmHg)	82	±	1	82	±	1	82	±	1	0.95	82	±	1	82	±	2	0.85	82	±	1	82	±	1	0.84
Glucose (mg/dL)	99.9	±	1.0	100.0	±	1.3	99.8	±	1.5	0.92	99.8	±	1.1	100.9	±	3.1	0.75	100.3	±	1.4	99.4	±	1.4	0.64
HbA_1c_ (%)	5.48	±	0.04	5.48	±	0.06	5.47	±	0.05	0.85	5.49	±	0.04	5.37	±	0.13	0.40	5.53	±	0.06	5.40	±	0.04	0.07
TG (mg/dL)	127	(93–171)		122	(92–169)		136	(102–179)		0.52	123	(92–171)		143	(121–166)		0.38	131	(94–166)		123	(92–189)		0.91
AST (IU/L)	23.0	(20.0–28.0)		22.0	(20.0–28.0)		24.0	(20.0–30.0)		0.35	22.0	(20.0–28.5)		25.5	(23.3–27.8)		0.49	23.5	(20.0–28.3)		23.0	(20.0–28.0)		1.00
ALT (IU/L)	28.0	(21.0–42.0)		27.0	(20.0–41.0)		30.0	(22.5–45.5)		0.32	28.0	(20.5–41.5)		29.0	(25.5–43.0)		0.53	28.0	(20.0–43.8)		27.0	(22.0–39.0)		0.87
γ-GTP (IU/L)	42.5	(31.8–58.9)		40.8	(30.1–55.1)		48.6	(34.4–77.6)		0.11	42.5	(32.3–59.2)		45.4	(26.7–53.8)		0.73	41.5	(32.5–57.7)		46.1	(29.6–61.5)		0.75
HDL-C (mg/dL)	50	±	1	49	±	1	52	±	2	0.18	50	±	1	47	±	4	0.52	50	±	1	50	±	2	0.86
LDL-C (mg/dL)	133	±	3	133	±	3	134	±	5	0.80	132	±	3	144	±	8	0.16	131	±	3	137	±	4	0.24
Total-C (mg/dL)	213	±	3	210	±	4	218	±	4	0.18	212	±	3	219	±	8	0.47	211	±	4	215	±	4	0.48
Lactate threshold (mL/kg/min)	16.6	±	0.2	16.7	±	0.3	16.5	±	0.3	0.68	16.6	±	0.2	16.5	±	0.8	0.94	16.5	±	0.3	16.7	±	0.3	0.56
Lactate threshold (METs)	4.74	±	0.05	4.76	±	0.07	4.72	±	0.08	0.68	4.74	±	0.05	4.73	±	0.22	0.94	4.72	±	0.08	4.78	±	0.07	0.56

Values are the mean ± SE or the median (interquartile range). PPAR, peroxisome proliferator-activated receptor; PRKAA2, α2 isoform of catalytic subunit of AMP-activated protein kinase; SNP, single-nucleotide polymorphism; BMI, body mass index; SBP, systolic blood pressure; DBP, diastolic blood pressure; HbA_lc_, hemoglobin A_lc_; TG, triglyceride; AST, aspartate aminotransferase; ALT, alanine aminotransferase; GTP, glutamyl-transpeptidase, HDL-C, high-density lipoprotein cholesterol; LDL-C, low-density lipoprotein cholesterol; Total-C, total cholesterol; METs, metabolic equivalents. * *p* value for between-group comparison using Welch’s t test (for age, weight, BMI, waist circumference, SBP, DBP, glucose, HbA_1c_, HDL-C, LDL-C, Total-C, lactate threshold) or Wilcoxon’s signed-rank test (for TG, AST, ALT, γ-GTP).

**Table 2:** Changes in the measured variables according to genotype groups of PPAR-δ, PPAR-γ, and PRKAA2 SNPs.

Variables	All Subjects (*n* = 109)			PPAR-δ rs2267668							PPAR-γ rs1801282							PRKAA2 rs1418442						
				A/A (*n* = 66)			A/G + G/G (*n* = 43)			*p* Value ^#^	C/C (*n* = 99)			C/G + G/G (*n* = 10)			*p* Value ^#^	A/A (*n* = 64)			A/G + G/G (*n* = 45)			*p* Value ^#^
Weight (kg)	−1.1	±	0.3 *	−0.1	±	0.3	−2.5	±	0.5 *	0.00	−1.2	±	0.3 *	0.8	±	1.2	0.13	−1.2	±	0.4 *	−0.9	±	0.5	0.58
BMI (kg/m^2^)	−0.36	±	0.10 *	−0.04	±	0.10	−0.84	±	0.18 *	0.00	−0.42	±	0.10 *	0.24	±	0.40	0.14	−0.40	±	0.14 *	−0.30	±	0.15	0.62
Waist circumference (cm)	−1.3	±	0.3 *	−0.6	±	0.3	−2.2	±	0.6 *	0.02	−1.4	±	0.3 *	−0.1	±	1.2	0.33	−1.4	±	0.4 *	−1.0	±	0.4 *	0.56
Glucose (mg/dL)	−0.4	±	0.8	0.2	±	1.0	−1.4	±	1.3	0.30	−0.1	±	0.8	−3.6	±	2.3	0.18	0.3	±	1.0	−1.5	±	1.3	0.27
HbA_1c_ (%)	−0.03	±	0.02	−0.002	±	0.03	−0.08	±	0.02 *	0.04	−0.03	±	0.02	0.00	±	0.05	0.51	−0.04	±	0.03	−0.02	±	0.03	0.79
TG (mg/dL)	−12.0	(−46.0–18.0) *		−2.5	(−33.5–21.8)		−33.0	(−59.0–−5.5) *		0.00	−12.0	(−48.5‒16.0) *		−21.5	(−26.5–29.0)		0.63	−20.0	(−42.8–10.8)		−8.0	(−58.0–22.0)		0.54
AST (IU/L)	−1.0	(−5.0–3.0) *		0	(−5.8–3.8)		−3.0	(−5.0–2.0) *		0.15	−1.0	(−5.0–2.5) *		−1.0	(−4.3–3.8)		0.61	−1.0	(−5.0–2.3)		−1.0	(−5.0–3.0)		0.78
ALT (IU/L)	−3.0	(−12.0–4.0) *		−0.5	(−8.8–4.0)		−7.0	(−12.5–1.5) *		0.02	−3.0	(−12.0–3.5) *		−3.5	(−8.5–3.3)		0.82	−2.0	(−11.3‒4.0) *		−5.0	(−12.0–3.0)		0.90
γ-GTP (IU/L)	−4.4	(−12.4–2.5)		−1.2	(−7.6–4.8)		−10.7	(−19.3–−2.5) *		0.00	−4.4	(−12.5–2.4)		−3.4	(−5.3–6.2)		0.52	−4.5	(−8.8–2.4)		−3.9	(−14.9–2.5)		0.35
HDL-C (mg/dL)	1.8	±	0.7 *	1.2	±	0.8	2.8	±	1.1 *	0.26	2.0	±	0.7 *	0.1	±	2.8	0.52	1.5	±	0.8	2.3	±	1.1 *	0.57
LDL-C (mg/dL)	−4.1	±	2.3	−4.5	±	2.3	−3.5	±	4.6	0.86	−4.3	±	2.4	−1.8	±	8.2	0.77	−5.5	±	3.3	-2.1	±	2.9	0.44
Total-C (mg/dL)	−6.7	±	2.2 *	−4.0	±	2.4	−10.8	±	4.2 *	0.16	−7.3	±	2.4 *	−0.6	±	6.0	0.32	−9.5	±	3.1^*^	-2.7	±	3.1	0.12

Values are the mean ± SE or the median (interquartile range). Note that post-intervention assessment (at the second checkup) was performed several months after the subject completed the six-month intervention. PPAR, peroxisome proliferator-activated receptor; PRKAA2, α2 isoform of catalytic subunit of AMP-activated protein kinase; SNP, single-nucleotide polymorphism; BMI, body mass index; SBP, systolic blood pressure; DBP, diastolic blood pressure; HbA_lc_, hemoglobin A_lc_; TG, triglyceride; AST, aspartate aminotransferase; ALT, alanine aminotransferase; GTP, glutamyl-transpeptidase; HDL-C, high-density lipoprotein cholesterol; LDL-C, low-density lipoprotein cholesterol; Total-C, total cholesterol. * *p* < 0.05 for within-group comparison between the baseline checkup and second checkup using Wilcoxon’s signed-rank test. ^#^ *p* value for between-group comparison using Welch’s t test (for weight, BMI, waist circumference, glucose, HbA_1c_, HDL-C, LDL-C, Total-C) or Wilcoxon’s signed-rank test (for TG, AST, ALT, γ-GTP).

**Table 3:** The multiple linear regression analyses on the associations of PPAR-δ *A/G* SNP (rs2267668) with the changes in the four clinical measures.

Outcome Variables	β	SE	*p* Value
Change in HbA_1c_	−0.045	0.039	0.26
Change in log-transformed TG	−0.211	0.089	0.02
Change in ALT	−3.5	3.4	0.31
Change in γ-GTP	−9.5	4.6	0.04

PPAR, peroxisome proliferator-activated receptor; SNP, single-nucleotide polymorphism; HbA_1c_, hemoglobin A_1c_; TG, triglyceride; ALT, alanine aminotransferase; GTP, glutamyl-transpeptidase.

## Supplementary Materials

The following are available online at http://www.mdpi.com/1422-0067/19/3/703/s1.
