# Supplementary material for: Influence of Single-Nucleotide Polymorphisms in PPAR-δ, PPAR-γ, and PRKAA2 on the Changes in Anthropometric Indices and Blood Measurements through Exercise-Centered Lifestyle Intervention in Japanese Middle-Aged Men
Source: Int J Mol Sci. 2018 Mar 1;19(3):703. doi: 10.3390/ijms19030703 (PMC5877564; doi:10.3390/ijms19030703)
Supplement: Supplementary file 1 [file ijms-19-00703-s001.pdf]

Supplementary Table S1. Post-intervention values of anthropometric/biochemical parameters mesured at second checkup according to genotypes of PPAR-δ, PPAR-γ, and PRKAA2 SNPs.

**Variables**	**All subjects (*n* = 109)**	**PPAR-δ rs2267668**		**PPAR-γ rs1801282**		**PRKAA2 rs1418442**	
		**A/A (*n* = 66)**	**A/G + G/G (*n* = 43)**	**C/C (*n* = 99)**	**C/G + G/G (*n* = 10)**	**A/A(*n* = 64)**	**A/G + G/G (*n* = 45)**
Weight (kg)	74.3 ± 0.8	75.9 ± 1.1	71.8 ± 1.2	73.7 ± 0.8	79.9 ± 3.7	74.4 ± 1.0	74.1 ± 1.3
BMI (kg/m^2)	25.3 ± 0.2	25.7 ± 0.3	24.7 ± 0.4	25.1 ± 0.2	27.5 ± 0.9	25.4 ± 0.3	25.3 ± 0.3
Waist circumference (cm)	88.8 ± 0.6	89.7 ± 0.8	87.4 ± 0.9	88.3 ± 0.6	93.1 ± 2.8	89.1 ± 0.9	88.3 ± 0.9
SBP (mmHg)	127 ± 1	129 ± 2	123 ± 2	127 ± 1	127 ± 4	127 ± 2	127 ± 2
DBP (mmHg)	82 ± 1	84 ± 1	80 ± 2	82 ± 1	81 ± 3	82 ± 1	82 ± 1
Glucose (mg/dL)	99.5 ± 1.2	100.3 ± 1.6	98.4 ± 1.5	99.7 ± 1.2	97.3 ± 2.4	100.6 ± 1.6	97.9 ± 1.6
HbA$_{1c}$ (%)	5.45 ± 0.05	5.48 ± 0.07	5.39 ± 0.04	5.45 ± 0.05	5.37 ± 0.11	5.50 ± 0.07	5.37 ± 0.04
TG (mg/dL)	112 (86–150)	120 (99–168)	94 (72–136)	111 (86–150)	128 (98–154)	115 (86–151)	109 (87–150)
AST (IU/L)	22.0 (19.0–25.0)	22.0 (19.0–26.0)	22.0 (18.0–24.0)	22.0 (18.5–24.5)	25.5 (22.0–27.8)	22.0 (19.0–24.3)	22.0 (19.0–26.0)
ALT (IU/L)	24.0 (19.0–33.0)	25.0 (20.0–35.0)	23.0 (17.5–31.0)	24.0 (19.0–32.0)	33.5 (20.3–38.0)	23.5 (19.0–34.0)	25.0 (19.0–31.0)
γ-GTP (IU/L)	36.9 (25.6–54.5)	38.8 (27.2–56.4)	35.2 (24.1–52.1)	36.9 (25.8–53.8)	39.6 (23.8–62.9)	36.2 (25.9–57.3)	37.2 (23.9–53.6)
HDL-C (mg/dL)	52 ± 1	50 ± 2	54 ± 2	52 ± 1	47 ± 2	51 ± 1	52 ± 2
LDL-C (mg/dL)	129 ± 3	128 ± 4	130 ± 4	128 ± 3	143 ± 8	125 ± 4	135 ± 4
Total-C (mg/dL)	206 ± 3	206 ± 4	207 ± 4	205 ± 3	218 ± 10	202 ± 4	213 ± 4

Values are the mean ± SE or the median (interquartile range). PPAR, peroxisome proliferator-activated receptor; PRKAA2, *α*2 isoform of catalytic subunit of AMP-activated protein kinase; SNP, single-nucleotide polymorphism; BMI, body mass index; SBP, systolic blood pressure; DBP, diastolic blood pressure; HbA$_{1c}$, hemoglobin A$_{1c}$; TG, triglyceride; AST, aspartate aminotransferase; ALT, alanine aminotransferase; GTP, glutamyl-transpeptidase, HDL-C, high descity lipoprotein cholesterol; LDL-C, low density lipoprotein cholesterol; Total-C, total cholesterol.
